# What Goes Around, Comes Around – HSV-1 Replication in Monocyte-Derived Dendritic Cells

**DOI:** 10.3389/fmicb.2017.02149

**Published:** 2017-11-07

**Authors:** Linda Grosche, Mirko Kummer, Alexander Steinkasserer

**Affiliations:** Department of Immune Modulation, University Hospital Erlangen, Erlangen, Germany

**Keywords:** HSV-1, dendritic cells, replication, cell-to-cell spread

## Abstract

HSV-1 is a very successful human pathogen, known for its high sero-prevalence and the ability to infect a wide range of different cell types, including dendritic cells (DCs). As very potent antigen-presenting cells DCs play an important role in the induction of antiviral immune responses and therefore represent a strategic target for viral-mediated immune escape mechanisms. It is known that HSV-1 completes its gene expression profile in immature as well as in mature DCs, while lytic infection is only found in immature DCs (iDCs). Notably, HSV-1 infected mature DCs (mDCs) fail to release infectious progeny virions into the supernatant. Apart from HSV-1 dissemination via extracellular routes cell-to-cell spread counteracts a yet unknown mechanism by which the virus is trapped in mDCs and not released into the supernatant. The dissemination in a cell–cell contact-dependent manner enables HSV-1 to infect bystander cells without the exposure toward the extracellular environment. This supports the virus to successfully infect the host and establish latency. In this review the mechanism of HSV-1 replication in iDCs and mDCs and its immunological as well as virological implications, will be discussed.

## Introduction

Herpesviruses comprise a family of large double-stranded DNA viruses and three subfamilies, i.e., α-, β- and γ-herpesviruses, are subdivided according to their cell tropism, pathogenesis, and genomic structure (Davison, 2007). Particular characteristics of these viruses are the tripartite gene expression cycle, consisting of an immediate early, early, and late phase as well as the ability to establish latent infection with lifelong persistence in the host (Wagner et al., 1995). Herpes simplex virus type I (HSV-1) is the prototypic α-herpesvirus with a sero-prevalence of 30% up to more than 90%, depending on age, socio-economic condition and geographical region (Nahmias et al., 1990; reviewed in Smith and Robinson, 2002). In acute infections HSV-1 usually causes cutaneous and mucosal herpes lesions, while severe symptoms, such as encephalitis or keratoconjunctivitis, are rarely observed. Although an antiviral immune response is successfully mounted, the host is unable to completely eliminate the virus. This is mainly achieved by infection and persistence in the trigeminal ganglion, an immune privileged niche, with subsequent establishment of a latent infection. Under certain circumstances, i.e., when the immune control is suboptimal, the virus reactivates, again leading to symptoms of acute infection (Whitley and Roizman, 2001).

All members of *herpesviridae* share a similar composition of enveloped virions, consisting of the viral dsDNA genome packed in the nucleocapsid, which is in turn coated by the inner as well as outer tegument, and the enclosing envelope containing viral encoded (glyco-) proteins (Roizman et al., 2007). During lytic replication, nucleocapsids assemble in the nucleus of the host cell and subsequently reach the nuclear membrane along actin fibers. In a process called nuclear egress the capsids first bud through the inner nuclear membrane into the perinuclear space, acquiring their primary envelopment. Due to their size the capsids are unable to cross the nuclear membrane via the nuclear pores. Therefore, the inner lamin layer has to be partially dissolved, which is mainly induced by viral/cellular kinases, resulting in pores allowing the transfer of the capsids (Mou et al., 2008; Lye et al., 2017). After fusion and translocation through the outer nuclear membrane, de-enveloped capsids are released into the cytoplasm. There, inner tegument proteins gather around the capsid while capsid-distal tegument proteins assemble at the final envelope site in the trans-Golgi network together with glycoproteins. Finally, mature virions are transferred to the plasma membrane and can either be released into the supernatant or passed on to adjacent cells in a cell–cell contact-dependent manner (reviewed in Mettenleiter et al., 2006).

Dendritic cells (DCs) constitute a group of leukocytes operating at the interface of innate and adaptive immunity, with the unique ability to activate naïve T lymphocytes (Steinman and Banchereau, 2007). Under homeostatic conditions, DCs reside immobilized in an immature state in peripheral tissues, possessing high phagocytic, but low T cell priming, capacity (Banchereau et al., 2000). Upon activation by stimulation via, e.g., pattern recognition receptors, antigen uptake or specific pro-inflammatory cytokines, immature DCs (iDCs) undergo a maturation process (Wilson and Villadangos, 2004). These maturing/mature DCs (mDCs) enhance their capacity of antigen processing and presentation, accompanied by increased major histocompatibility complex (MHC) class I and class II expression levels together with upregulation of costimulatory molecules of the B.7 family (CD80 and CD86) as well as CD40 (Banchereau and Steinman, 1998; Watts et al., 2007). Furthermore, changes in the surface molecule repertoire important for interactions with other immune cells occur, since mDCs upregulate, e.g., expression of intercellular adhesion molecule 1 (ICAM-1, CD54) and CD83 (Zhou and Tedder, 1996; Banchereau and Steinman, 1998; Prechtel et al., 2007). Upon maturation DCs further undergo a switch in their chemokine receptor expression profile, amongst others important for migration, primarily to areas of high T-lymphocyte density in secondary lymphoid organs, via the CCR7-CCL19 axis (reviewed in Sallusto et al., 1998; Banchereau et al., 2000; Ohl et al., 2004). Considering that DCs are essential inductors of antiviral immune responses by activating T-cell mediated immunity, they represent interesting immune evasion targets for invading pathogens, and especially for herpesviruses, which have been shown to modulate vital DC functions.

All of the clinical manifestations of an HSV-1-infection are a result of the ability to spread from the initially infected to uninfected bystander cells in primary as well as recurrent infections. Infection of host cells by HSV-1 is initiated by interaction of viral envelope proteins with cellular surface receptors (Spear and Longnecker, 2003). Depending on the respective cell type, different cellular surface receptors are known to be essential for virion attachment and entry. Regarding DCs, it was shown that in cell-free infection specific glycoproteins of the HSV-1 envelope bind to the host cell’s DC-specific ICAM-grabbing non-integrin (DC-SIGN), heparan sulfate proteoglycan, nectin-1/2, PILRα and the herpesvirus entry mediator (Salio et al., 1999; de Jong et al., 2008; Satoh et al., 2008). Interestingly, HSV-1 is able to switch between cell-free and cell-to-cell spread (reviewed in Sattentau, 2008). However, the latter is not completely understood in the context of DCs until now, but might be of high relevance for viral spread and therefore pathogenicity.

This review focuses on the interplay between immature as well as mature monocyte-derived DCs with HSV-1 regarding viral replication and spread.

## DCs As Targets for HSV-1 Mediated Immune Escape Mechanisms

During the interplay between HSV-1 and the host this virus evolved several immune escape mechanisms counteracting antiviral immune responses. For the induction of protective antiviral immunity, DCs play an essential role by activating antigen-specific T cells (Sprecher and Becker, 1986; Allan et al., 2003). Thus, manipulation of DC functions by HSV-1 enables the establishment of successful viral replication and latency. In primary infections HSV-1 initially replicates in infected epithelial cells, including epidermal keratinocytes (Nicola et al., 2005). For migration to the nervous system, newly produced progeny virions subsequently infect adjacent cells at the site of primary infection, including DCs (Becker, 2002). In the first line predominantly iDCs but also mature bystander DCs (mDC), that matured upon specific signals released by, e.g., infected keratinocytes or iDCs, can be infected (Mikloska et al., 1998; Palucka and Banchereau, 2002). Once having access to these essential antigen presenting cells, the virus exhibits a variety of immune evasion strategies that have been developed during millions of years of co-evolution. Typical examples are the downmodulation of surface molecules such as CD83, CCR7, CXCR4, or IFNGR1 (Kruse et al., 2000; Prechtel et al., 2005; Kummer et al., 2007; Eisemann et al., 2008; Theodoridis et al., 2011; Heilingloh et al., 2014).

## HSV-1 Replication in Immature DCs

**FIGURE 1:**
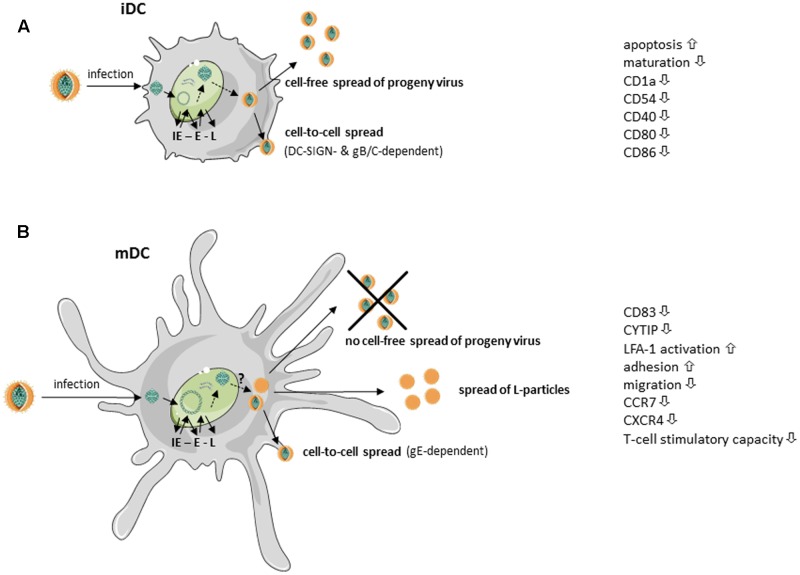
Simplified scheme of HSV-1 replication in immature and mature dendritic cells (taken and modified from SMART servier medical art). HSV-1-infected **(A)** immature dendritic cells (iDCs) as well as **(B)** mature DCs (mDCs) exhibit immediate early (IE), early (E), and late (L) expression of viral gene products. **(A)** HSV-1-infected iDCs release fully matured HSV-1 virions into the supernatant. Additionally, HSV-1 spreads in a gB/gC- and DC-specific ICAM-grabbing non-integrin (DC-SIGN)-dependent manner to adjacent cells. Surface expression of, e.g., CD1a, intercellular adhesion molecule 1 (ICAM-1, CD54), CD40, CD80 and CD86, is reduced on HSV-1-infected iDCs. Furthermore, HSV-1 inhibits iDC maturation and induces apoptosis. **(B)** Fully mature HSV-1 virions spread in a gE-dependent manner from infected mDCs to adjacent cells. HSV-1 inhibits DC migration by downregulation of cytohesin-1 interacting protein (CYTIP), thereby increasing mDC adhesion via induction of lymphocyte function-associated antigen 1 (LFA-1) activity. Surface expression of the chemokine receptors CCR7 as well as CXCR4 and the maturation marker CD83 are downmodulated on HSV-1-infected mDCs. However, HSV-1-infected mDCs are unable to release fully matured virions into the supernatant. In addition, light (L)-particles, lacking the capsid, are generated which are able to modulate uninfected bystander cells.

HSV-1 can successfully infect both iDCs and mDCs, however, replication occurs in an unequal fashion (Mikloska et al., 2001; Goldwich et al., 2011). When HSV-1 infects iDC, the virus completes its replication cycle, cells are lysed and progeny virus is released into the supernatant (Mikloska et al., 2001). Prior to lysis, HSV-1 infected iDCs downregulate surface expression of adhesion as well as costimulatory molecules, including CD1a, CD40, ICAM-1 (CD54) and later during infection CD80 and CD86 (Mikloska et al., 2001). Moreover, HSV-1 inhibits maturation of infected iDCs – very likely via ICP34.5 – and therefore interferes with upregulation of various surface molecules involved in the induction of potent antiviral immune responses (Salio et al., 1999; Ma et al., 2017). The newly generated and released virions can subsequently infect bystander cells, DCs or other cell types, in a cell-free manner. This mode of viral transmission enables the virus to infect a high number of adjacent cells and to disseminate rapidly via lymph, blood, or cerebrospinal fluid (Sattentau, 2008). Apart from this cell-free spread from infected iDCs, HSV-1 was further shown to disseminate also in a cell-to-cell dependent manner. Work from de Jong et al. (2008) revealed the internalization and cell–cell contact-dependent transmission of HSV-1 via iDCs to be DC-SIGN-dependent. While the viral glycoproteins gB and gC were shown to be relevant, the authors point out that further viral proteins, such as gE or gD, could additionally be important. However, the lysis of HSV-infected iDCs not only leads to the release of progeny virus but also to the secretion of a variety of cytokines, some of them delivering “danger” signals to surrounding iDCs triggering their maturation. This together with the uptake of cell debris from apoptotic/lysed cells allows cross-presentation of virus-derived antigens to efficiently stimulate cytotoxic T cells (Bosnjak et al., 2005). These different mechanisms are summarized in **Figure 1A**.

## HSV-1 Replication in Mature DCs and Cell-to-Cell Spread

Previously, it has been postulated that HSV-1 is not able to complete its replication cycle in mDCs. This seemed reasonable due to an absence of infectious viral particles in the supernatant of HSV-1-infected mDCs (Kruse et al., 2000). However, our group was later able to show, that HSV-1 completes its replication cycle also in mDC, since both transcripts as well as proteins of all three phases of the viral replication cascade could be detected in infected mDC. As transition from one phase to the successive phase is dependent on the proteins of the preceding phase, the presence of late proteins confirms that the cascade has indeed been completed (Goldwich et al., 2011; see also **Figure 1B**). The question why progeny virus is only very poorly released into the supernatant of infected mDCs is still under investigation.

Interestingly, our group provided evidence that progeny virus of infected mDCs can be passed on to adjacent cells in a cell–cell contact-dependent manner. This mechanism was proven to be dependent on the viral glycoprotein E, as the respective HSV-1 deletion mutant failed to infect adjacent permissive cells (Goldwich et al., 2011). Earlier, HSV-1 cell-to-cell spread was shown to be amongst others dependent on the viral glycoproteins gI and gE, since gI/gE mutants showed reduced spread between cultured epithelial and neuronal cells. For proper trafficking of HSV-1 particles to other cells, the gI/gE complex has to accumulate in the trans-Golgi network (TGN) during the early phase of infection. Subsequently, gI/gE is specifically sorted to cell junctions in the late phases (Balan et al., 1994; Dingwell and Johnson, 1998; McGraw and Friedman, 2009). Johnson and colleagues demonstrated the cytoplasmic domain of gE to be indispensable for TGN localization of gI/gE, as mutants spread poorly and showed similar defects as a gE null mutant (Polcicova et al., 2005; Farnsworth and Johnson, 2006). Although less is known in respect to the cell–cell contact-dependent spread of HSV-1 via mDCs, several HSV-1 proteins were found to interact with gE and therefore to play an essential role in viral cell-to-cell spread in other cells. Interestingly, Haugo et al. (2011) showed that the viral protein pUL34 is required for proper localization of gE at the plasma membrane and, more important, junctional surfaces in Vero cells. They demonstrated that gE is mislocated in cytoplasmic membrane aggregates, when Vero cells were infected with a pUL34-null virus. Furthermore, also the viral factor pUL51 has been shown to be important for gE trafficking, since it interacts with gE and directs its proper localization (Roller et al., 2014). Several other conserved herpesviral-encoded gene products were also implicated to influence proper gE function or localization in infected cells and therefore cell-to-cell spread, i.e., UL11, UL16, UL21, and UL49 (Duffy et al., 2006; Han et al., 2011, 2012). It is likely that the interplay of these viral gene products in cell-to-cell spread, reported recently, will also prove significant in mDCs.

Apart from the release of fully mature progeny virus, we recently reported the generation of L-particles by HSV-1-infected mDC (Heilingloh et al., 2015; see also **Figure 1B**). L-particles together with H-particles are generated during the lytic replication of HSV-1 in many cell types. While H-particles represent full virions, L-particles lack the capsids and the viral DNA and are therefore not infectious (McLauchlan and Rixon, 1992; Rixon et al., 1992). As previously published, the release of particles similar to L-particles from HSV-1 infected cells, can also be promoted by blocking DNA replication (Dargan et al., 1995). This report shows that the nature of the generated particles can be artificially shifted toward L-particle-like entities called PREPS (pre-viral DNA replication enveloped particles). Considering this, in mDC specific obstacles might impede the formation of fully mature virions, thereby inducing the release of L-particles. The underlying mechanisms are currently under investigation.

## Dissemination of HSV-1 via the Virological Synapse

Viruses use different ways to disseminate within the host, whereby cell-to-cell spread, in contrast to transmission by extracellular routes, offers the possibility to disseminate throughout the host without being easily recognized by the host’s immune system. Therefore, herpesviruses can spread in a cell–cell contact-dependent manner despite the presence of neutralizing antibodies or potent immune responses (Sattentau, 2008). Cell-to-cell spread from mDCs to other cells might be of high relevance considering the intimate interaction of mDC with T cells. Although HSV-1 is a rather non-lymphotropic virus, several lines of evidence suggest that HSV-1 can infect antigen-specific cytotoxic (activated) T cells (Nahmias et al., 1964; Kirchner et al., 1977; Teute et al., 1983; Raftery et al., 1999; Sloan and Jerome, 2007). Moreover, it has previously been shown that HSV-1 can be transferred from fibroblasts to T cells via a virological synapse-like structure. Infection of T cells via HSV-1-infected fibroblasts was significantly increased in this cell–cell contact-dependent manner compared to cell-free virus, and interestingly prior activation of T cells led to an even more pronounced permissivity. HSV-1 gE and gI were not required for cell-to-cell spread from fibroblasts to T cells, however gD was found to play an essential role (Aubert et al., 2009). Furthermore, HSV-1-infected T cells exhibited a modulated T-cell receptor signaling and altered cytokine production favoring an IL-10-rich and immunosuppressive milieu (Sloan et al., 2006; Sloan and Jerome, 2007), which can be considered as an escape mechanism to prevent the induction of proper immune responses. Interestingly, unpublished data from our group showed that HSV-1 is able to spread *in vitro* in a cell–cell contact-dependent manner from initially infected mDCs to T cells.

The virological synapse is a specialized structure used by certain lymphotropic viruses, e.g., human T-cell leukemia virus type 1 (HTLV-1) or human immunodeficiency virus (HIV) for infection of T cells (Gardiner et al., 2016; Gross and Thoma-Kress, 2016). The composition of the virological synapse is similar – but not identical – to a structure formed between antigen-presenting cells, e.g., DCs and T cells, called immunological synapse. More precisely, in both synapses the adhesion molecule lymphocyte function-associated antigen 1 (LFA-1) as well as the respective ligands ICAM-1 and ICAM-3 are highly enriched (Vasiliver-Shamis et al., 2010). It is tempting to speculate that the transfer of progeny HSV-1 from mDCs to T cells could be mediated via this structure and enhanced by HSV-1-mediated downmodulation of the cytohesin-interacting protein (CYTIP) in HSV-1 infected mDCs (Theodoridis et al., 2011). CYTIP counteracts cytohesin-1 mediated LFA-1 activation by binding to cytohesin-1, thereby interrupting the activation of LFA-1 (Boehm et al., 2003). Moreover, our group and others demonstrated that CYTIP regulates contact duration between DCs and T cells, since its downregulation ultimately led to increased adhesion and a prolonged contact with T cells (Hofer et al., 2006; Theodoridis et al., 2011). Supporting the hypothesis, that elevated LFA-1 activity could improve the contact and therefore formation of a virological synapse between DCs and T cells, Aubert et al. (2009) indeed showed an enhanced LFA-1 activity on T cells at virological synapse-like structures at the site of contact with HSV-1-infected fibroblast. Interestingly, viral spread via the virological synapse could be enhanced or inhibited by inducing or blocking LFA-1, respectively (Aubert et al., 2009). Apart from using this prolonged contact duration between DC and T cell as strategy for viral spread, T cells are unable to properly differentiate into cytotoxic effector cells. Hence, the antiviral T cell mediated immune response can be inhibited. Furthermore, loss of CYTIP protein levels as well as surface expression of the chemokine receptor CCR7 in HSV-1-infected mDCs leads to an inhibition of mDC migration toward, e.g., CCL19, highly expressed in secondary lymphoid organs. Thus, infected mDCs reside immobilized at the site of infection and are unable to activate T lymphocytes in lymphoid tissues (Prechtel et al., 2005; Theodoridis et al., 2011).

## Conclusion and Perspectives

HSV-1 is a very successful human pathogenic virus able to infect a wide range of different cell types, including the most potent antigen presenting cells, i.e., DCs. The infection of DCs with HSV-1 is characterized by viral modulations of vital DC functions in order to subvert their essential role in the induction of a potent antiviral immune response, and in turn to promote efficient viral replication, dissemination as well as latency.

Cell-to-cell spread is of high clinical relevance not only during primary infections, when the virus spreads from the initially infected cell retrograde for neuroinvasion, but also in recurrent infection, with anterograde spread of HSV-1 along axons (Enquist et al., 1998; Diefenbach et al., 2008). Considering that no vaccine is available to cure HSV-1 infections and given the concurrent increasing frequency of multidrug-resistant HSV strains in patients, there is an increasing need to better understand viral dissemination and entry into host cells. In this regard, cell-to-cell spread is of great interest, as the virus is able to hide from immunological barriers. Several aspects were considered to target and limit HSV-1 cell-to-cell spread; e.g., multivalent gold nanoparticles that mimic host cell heparan sulfate (Baram-Pinto et al., 2010), human lactoferrin (Välimaa et al., 2009), α- and γ-IFN (Mikloska and Cunningham, 2001) or anti-HSV monoclonal antibodies (Krawczyk et al., 2015). However, there is still a vital medical need for the development of new and better antiviral strategies.

## Author Contributions

All authors listed have made a substantial, direct and intellectual contribution to the work, and approved it for publication.

## Conflict of Interest Statement

The authors declare that the research was conducted in the absence of any commercial or financial relationships that could be construed as a potential conflict of interest.
